# Site‐dependent regulation of breeding success: Evidence for the buffer effect in the common guillemot, a colonially breeding seabird

**DOI:** 10.1111/1365-2656.13674

**Published:** 2022-02-25

**Authors:** Sophie Bennett, Sarah Wanless, Michael P. Harris, Mark A. Newell, Kate Searle, Jonathan A. Green, Francis Daunt

**Affiliations:** ^1^ UK Centre for Ecology & Hydrology Penicuik UK; ^2^ School of Environmental Sciences University of Liverpool Liverpool UK

**Keywords:** common murre, density dependence, habitat quality, population recovery, population resilience, public information, site quality, *Uria aalge*

## Abstract

1. Density‐dependent regulation can offer resilience to wild populations experiencing fluctuations in environmental conditions because, at lower population sizes, the average quality of habitats or resources is predicted to increase. Site‐dependent regulation is a mechanism whereby individuals breed at the highest quality, most successful, sites, leaving poorer quality, less successful sites vacant. As population size increases, higher quality sites become limiting but when populations decline, lower quality sites are vacated first, offering resilience. This process is known as the ‘buffer effect’. However, few studies have tested whether such regulation operates in populations experiencing changes in size and trend.
2. We used data from a population of common guillemots *Uria aalge*, a colonially breeding seabird, to investigate the relationship between site occupancy probability, site quality and population size and trend. These data were collected at five sub‐colonies spanning a 38‐year period (1981–2018) comprising phases of population increase, decrease and recovery.
3. We first tested whether site quality and population size in sub‐colonies explained which sites were occupied for breeding, and if this was robust to changes in sub‐colony trend. We then investigated whether disproportionate use of higher quality sites drove average site quality and breeding success across sub‐colony sizes and trends. Finally, we tested whether individuals consistently occupied higher quality sites during periods of decline and recovery.
4. Higher quality sites were disproportionality used when sub‐colony size was smaller, resulting in higher average site quality and breeding success at lower population sizes. This relationship was unaffected by changes in sub‐colony trend. However, contrary to the predictions of the buffer effect, new sites were established at a similar rate to historically occupied sites during sub‐colony decline and recovery despite being of lower quality.
5. Our results provide support for the buffer effect conferring resilience to populations, such that average breeding success was consistently higher at lower population size during all phases of population change. However, this process was tempered by the continued establishment of new, lower quality, sites which could act to slow population recovery after periods when colony size was low.

## Graphical abstract

As more populations face anthropogenic threats and experience changes in size and trend, there is an increasing need to understand species' ability to buffer against these pressures. This research shows that populations can maintain resilience as they fluctuate in size and trend through consistent occupation of the highest‐quality breeding sites.

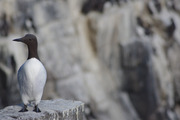

## 1 INTRODUCTION

Density‐dependent regulation of populations is a widespread phenomenon in natural systems, shaping the responses of populations to fluctuating or intensifying extrinsic pressures (Denley & Metaxas, 2017; Matte et al., 2020). Negative density dependence occurs when demographic rates such as productivity or survival increase at lower population density as a result of reduced competition for finite resources, such as breeding sites or food. As such, density‐dependent regulatory mechanisms may act to offer resilience to relatively small populations (Bottero et al., 2017). For critical resource types such as food (Ashbrook et al., 2010) and habitat (Lindberg et al., 2006), there is a good understanding of how density dependence affects resource use and demographic rates at different population sizes. However, there is more limited understanding of whether density dependence operates consistently in declining and increasing phases in fluctuating populations (Hoy et al., 2017; Korpimäki & Krebs, 1996). Population structure may differ between increasing and declining phases, in particular the proportion of new, inexperienced individuals, which may affect the dynamics of density dependence. With many wild populations experiencing declines as a result of multiple anthropogenic threats, it is vital to understand the relationship between density‐dependent resource limitation and subsequent consequences for demographic rates during all population trajectories.

Habitat can vary in quality, and, as a result, competition for habitat is a key density‐dependent regulatory mechanism that can offer resilience to population declines. Higher quality habitat may be preferentially occupied to, for example, increase access to resources (Morris & MacEachern, 2010) or provide shelter from harsh environmental conditions (Rodenhouse et al., 1997). Heterogeneity in habitat quality may subsequently contribute to variation in demographic rates such as survival (Paradis, 1995) and productivity (Denley & Metaxas, 2017) among individuals. As population size increases, higher quality habitat becomes limited, forcing individuals to occupy habitat of lower quality (Gill et al., 2001; Kokko et al., 2004). In contrast, when a population experiences a decline, individuals are predicted to disproportionately occupy higher quality habitats—a process known as the ‘buffer effect’ (Gill et al., 2001; Morris & MacEachern, 2010). Whether the buffer effect can operate across phases of population increase, decline and recovery will determine its capacity to consistently offer resilience (Rodenhouse et al., 1997). However, the precise behavioural mechanisms whereby higher quality habitats are occupied at lower population sizes may not operate as effectively in all population trends. Further, in order to disentangle stochastic annual variation in extrinsic factors such as prey abundance that may act to dampen or strengthen the buffer effect (Gill et al., 2001) (Gaston et al., 2007), long‐term data on habitat use across a range of population sizes and trends are required. However, to our knowledge, an explicit test of whether density‐dependent habitat use and associated effects on demographic rates are observed across all phases in a fluctuating population has not previously been carried out.

An important mechanism by which the buffer effect may shape demographic rates is through site‐dependent regulation of breeding success (Kokko et al., 2004; Soutullo et al., 2006). Where this process is operating, average breeding success decreases as the population expands into breeding sites of lower quality (Kokko et al., 2004; Rodenhouse et al., 1997). Conversely, average breeding success increases when a population is declining, as remaining individuals occupy nest sites of higher quality (Sullivan et al., 2015). A critical process that will determine the efficacy of the buffer effect is the means by which individuals are able to assess site quality, for example through individual detection (Espie et al., 2004) or the use of public information (Doligez et al., 2002). Such mechanisms potentially inform individuals about the best available sites given the prevailing population size and associated conspecific competition. As populations decline, surviving individuals may have accurate information on site quality, and can benefit from the greater availability of high‐quality sites. In contrast, as populations increase, there will be a higher proportion of naïve individuals that may lack the ability or experience to assess site quality accurately. For example, such individuals may be more likely to select new sites that are of lower average quality than those that have been used historically (Rodenhouse et al., 1997; Soutullo et al., 2006). As such, density‐dependent effects on site occupancy may be modulated by population trend due to variation in the relative number of naïve individuals. Consequently, different patterns of change in site occupancy and average quality during different phases of growth in fluctuating populations may occur. In particular, studies have not tested the rate of establishment of new sites versus re‐colonization of previously used sites during population recovery, and what effect this may have on average site quality and, ultimately, breeding success. This is critical because the ability of individuals within a recovering population to identify sites of higher quality may strongly affect the speed at which a population recovers from a decline.

Here we investigated whether site‐dependent regulation operates consistently across population sizes and trends in a population of a colonial seabird species, the common guillemot *Uria aalge* (hereafter, guillemot). The guillemot is one of the most densely breeding bird species with records of >70 nest sites per m^2^ (Birkhead, 2010). While birds may change nest sites, most birds at the study colony retain the same nest site from year to year (Harris et al., 1996). Site‐dependent regulation has previously been demonstrated in the study population such that the highest quality sites were more likely to be occupied and the average quality of nest sites decreased as the breeding population increased in size (Kokko et al., 2004). However, the study was undertaken during a period of population growth (1981–2002). Subsequently, the breeding population declined (2002–2013) and then entered a period of recovery (2013–2018). These fluctuations in population size and growth trajectories provide a useful opportunity to extend the approach of Kokko et al. (2004) and test whether site‐dependent regulation is consistent across population trends.

**FIGURE 1:**
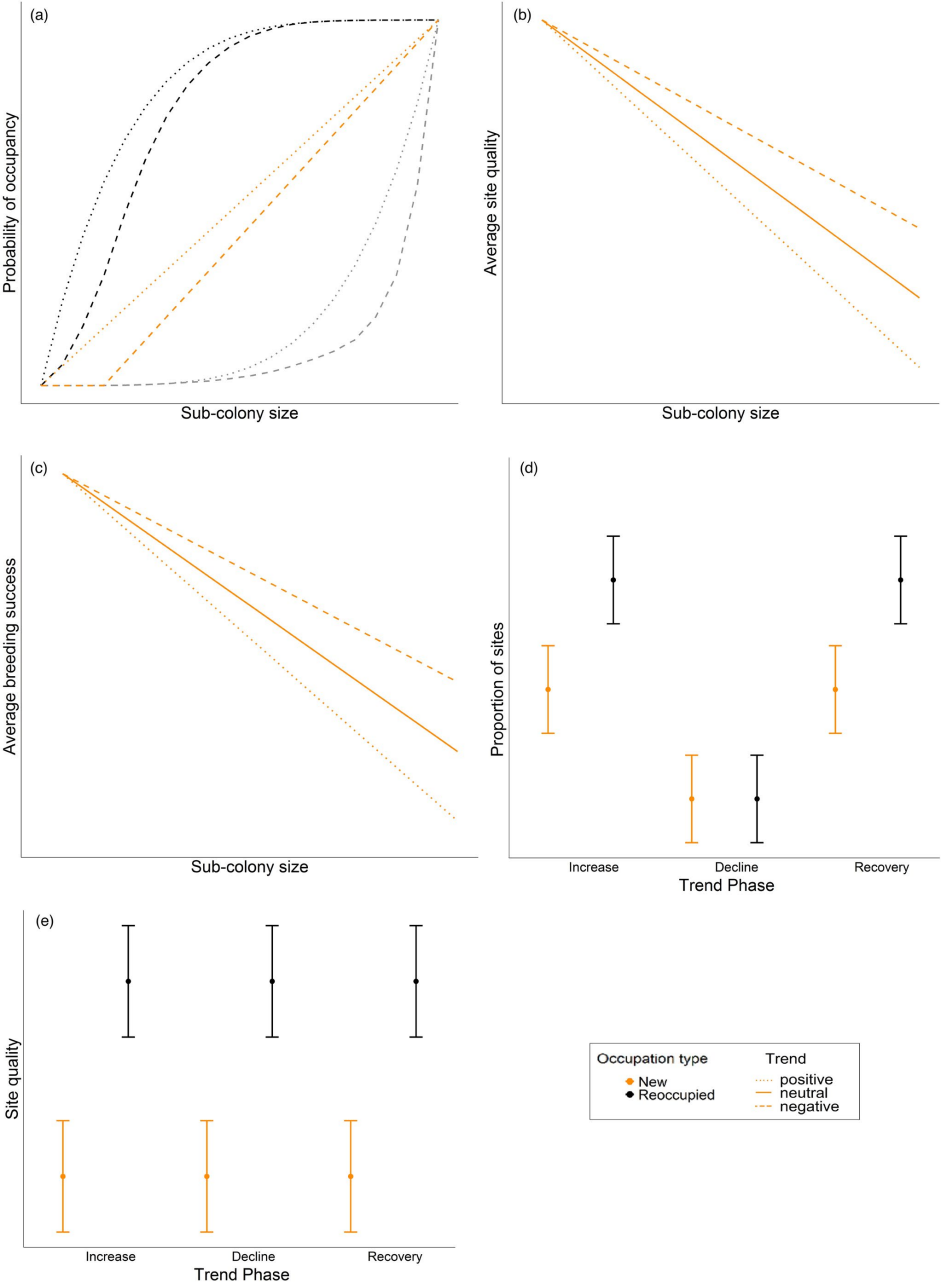
Schematic of site occupancy and quality hypothesis predictions. (a) shows the interactive effect of sub‐colony size and site quality on site occupancy, and how it may change under different sub‐colony trends (Hypothesis 1). (b) and (c) show the effect of sub‐colony size on average site quality and average breeding success, respectively, and how this is modulated by sub‐colony trends (Hypotheses 2 and 3). (d) and (e) show the predicted effect of trend phase (Hypotheses 4a and b) and site quality (Hypothesis 4c) on the occupation of new and reoccupied sites respectively. In (a)–(c) site quality is indicated by colour, and sub‐colony trend by line type: black (high quality), orange (average quality), grey (low quality), solid lines (no change in sub‐colony trend), dashed lines (negative sub‐colony trend) and dotted lines (positive sub‐colony trend). In (d) and (e), colour indicates whether sites were new (orange) or reoccupied (black)

We tested four hypotheses associated with the effect of population size and trend on site quality, probability of occupancy and breeding success, and controlled for population‐level extrinsic factors that may also affect the population in a density‐dependent manner (Table 1; Figure 1). Overall, we predicted that this population is subject to site‐dependent regulation but there may be a lack of consistency in the strength of this regulatory process depending on population trend (increasing and declining) and phase (increasing, declining and recovering).

**TABLE 1:** Hypotheses and predictions of the relationship between site quality, occupancy and population size and trend

Hypothesis	Prediction
H1: Sites of high quality will be more likely to be occupied, particularly at lower population sizes, and this relationship will vary with population trend	a) High‐quality sites are more likely to be occupied than low‐quality sites at all population sizes b) The effect of site quality will be greater at lower population sizes. c) The effect of site quality and population size on site occupancy will intensify under more positive population trends.
H2: Average site quality will increase at lower population sizes and this relationship will vary with population trend	a) If H1 is supported, the average quality of occupied sites will be higher at lower population sizes when a smaller proportion of lower quality sites are occupied b) The decline in average quality of occupied sites with increasing population size will intensify under more positive population trends
H3: Average breeding success will be higher at lower population sizes and this relationship will vary with population trend	a) If H1 and H2 are supported, average breeding success will be higher at lower population sizes b) The decline in average breeding success of occupied sites with increasing population size will intensify under more positive population trends
H4: The rate of establishment of new sites will depend on population trend phase	a) More new sites will be established in positive trend phases (increase and recovery), compared to negative phases (declines) b) During positive trend phases, historically occupied sites will be more likely to be occupied than new sites c) Reoccupied sites will be of higher quality than new sites, and this will drive H4b

## 2 MATERIALS AND METHODS

### 2.1 Study population

The study was undertaken on the Isle of May National Nature Reserve in south‐east Scotland (56′11″N, 2′33″W) from 1981 to 2018 under licence from NatureScot (licence MON/RP/181 and its **predecessors**). Guillemots breed on the cliffs between April and July and breeding sites constitute a bare area of rock ~10 cm in diameter. Guillemots have a single egg clutch but may replace eggs that are lost early in the breeding season (Harris & Wanless, 1988). The breeding success and population size of guillemots were studied annually. Breeding sites were monitored up to four times daily throughout the breeding season at five sub‐colonies from permanent hides (mean number of sites/sub‐colony = 171, range = 37–323). The boundaries of these sub‐colonies remained constant throughout the study period and all had capacity for additional breeding pairs at the start of the study. The sub‐colonies were located on the west side of the island within 100 m of each other and were selected to cover the full range of breeding habitats and site types used by guillemots at this colony, that is habitats varying in their physical characteristics (wide flat ledges with 100+ pairs, narrow ledges of varying slopes with few pairs, exposed platforms and enclosed ‘niches’; full range of heights above sea level and aspect). Monitoring commenced at two of the sub‐colonies in 1981, at one in 1983 and at two in 1984 (Harris & Wanless, 1988). The number of breeding pairs in the whole colony was estimated annually from 1984, following the methods described in Harris et al. (2015).

Each breeding site in each sub‐colony was assigned a unique identification number in the first year it was occupied (i.e. when an egg as laid) and was mapped on photographs to allow consistent monitoring between the years. At each site, egg laying and chick fledging success were recorded. A chick was considered to have fledged successfully if it reached an age of 15 days unless there was any evidence to the contrary (Harris et al., 2020). The number of occupied breeding sites in each sub‐colony (hereafter ‘sub‐colony size’) was recorded each year.

### 2.2 Data treatment

#### 2.2.1 Estimating site quality

Previous studies of this population have estimated site quality in two ways: the physical characteristics of a breeding site and the historic breeding success at the site (i.e. average breeding success in past years; Harris et al., 1997; Kokko et al., 2004). Harris et al. (1997) found that six physical characteristics had a significant relationship with average breeding success. Historical breeding success was also strongly correlated with current breeding success (Harris et al., 1997; Kokko et al., 2004). Measures of physical characteristics were not available for sites in our study that were first occupied after 2008 (*n* = 414/1664) but data were available on breeding success. Following Kokko et al. (2004), we therefore used the average breeding success at sites across all the years as our measure of site quality. To confirm that the relationship between breeding success and physical site characteristics still held with the inclusion of more years and sites, we repeated the analysis undertaken by Harris et al. (1997) and Kokko et al. (2004). We found that the principal components derived from the physical characteristics showed a strong relationship with average breeding success at the site. We were therefore confident that average breeding success is an appropriate measure of site quality (full details in Supplementary 1).

### 2.3 Quantifying trends in sub‐colony and whole colony size

We considered sub‐colony to be the most appropriate scale for testing the effect of population size on site occupancy because (a) prior to breeding for the first time, most individuals spend one or two seasons at or near the sub‐colony where they later breed (Halley et al., 1995) and (b) once they have bred, most birds retain the same nest site each year while the few that do change site typically move less than 2 m (Harris et al., 1996).

However, we also tested whether there was density‐dependent site occupancy and breeding success related to extrinsic factors such as food supply, which is decoupled from site quality and acts on the whole population. To do this, we quantified the equivalent measures of trends in population size for both sub‐colonies and the whole colony.

To quantify the trends in population size in sub‐colonies, we first partitioned each time series of sub‐colony size into multi‐year periods of consistent population trend direction. We used the r package ‘strucchange’ (Zeileis et al., 2002, 2003) to identify the year(s) when the population trend significantly changed in direction (break points) and categorized periods either side of break points as either ‘increasing’, ‘stable’ or ‘decreasing’. We considered the year when a break point occurred as the first year in the next trend period. For each of these periods, we then calculated the average annual change in sub‐colony size, termed ‘sub‐colony trend’, which we used in all analyses for Hypotheses 1–3 (see Supplementary 2 for sub‐colony trends). We then repeated this process using whole colony population size. We adopted this trend‐based approach in preference to quantifying year‐to‐year changes which are more likely to be affected by environmental stochasticity as opposed to reflecting true changes in population size (Reed et al., 2015). This was of particular importance in this study where for some tests we used trends categorized on their direction alone.

To test whether the number of new sites changed with sub‐colony trend phase (Hypothesis 4), we classed each phase as either ‘increasing’ (positive sub‐colony trend) or ‘decreasing’ (negative sub‐colony trend). We then partitioned these phases into increasing phases (‘increase’), ‘decreasing’ phases (‘decline’) and increasing phases following a decline (‘recovery’). Again, we repeated this procedure using the data for whole colony population size. Each sub‐colony and the colony as a whole experienced a sequence of one or more increasing phases, then one or more declining phases and then a recovery phase.

### 2.4 Identifying new and historically occupied (reoccupied) sites

To determine whether historically occupied sites were reoccupied at a higher rate and were of higher quality than new sites (Hypotheses 4b and c), we defined reoccupied sites as those that had been occupied in a previous phase, were not occupied in the first year of the current phase but were later occupied in the current phase. To estimate the rate of reoccupation in the first increasing phase, we used the first 5 years as a ‘burn‐in’, because a small proportion of guillemots in this population may skip breeding for one or more years (Reed et al., 2015; average annual skipping frequency = 7.1% [*n* = 696/9,741 individuals]). As such, we minimized the possibility that we would incorrectly classify reoccupied sites as new sites. We considered sites to be reoccupied if they had been occupied at least once in the burn‐in period, were unoccupied in the sixth year and then occupied later in the increasing phase. To ensure comparability with new sites, we only considered new sites from the sixth year in this phase. We then calculated the proportion of sites that were new and reoccupied out of the total number of sites occupied in each sub‐colony in each year, to account for size differences between sub‐colonies.

### 2.5 Statistical analysis

We used general and generalized linear mixed‐effects models to test four hypotheses of site‐dependent regulation. All models had the following general structure; see further details below for where models deviated from this:

$$\gamma_{\left[t,c,s\right]}\sim {\text{Binomial}}_{\text{logit}}\;\left(\alpha +\sum \beta_{i}\cdot X_{\left[i,t,c,s\right]}+\varepsilon_{y}+\varepsilon_{c}+\varepsilon_{s}\right),$$

where *γ*_[*t*,*c*,*s*]_ is the response for year ‘*t*’ in sub‐colony ‘*c*’ at site ‘*s*’, *α* is the intercept, *β*_*i*_ is the slope coefficient for the fixed effect of explanatory variable *Χ*_[*i*]_ at site ‘*s*’ in sub‐colony ‘*c*’ and *ε*_*y*_ + *ε*_*c*_ + *ε*_*s*_ are random effects for year, sub‐colony and site.

In all models we included a random intercept of year to account for year‐to‐year variation in stochastic extrinsic factors. We also included a random intercept for sub‐colony to account for differences in occupancy and quality between sub‐colonies. Where we had multiple measures for sites, we included a nested random intercept of site within sub‐colony. We also included fixed effects for whole colony size and trend equivalent to the sub‐colony measures, for example if a model included fixed terms of sub‐colony size and sub‐colony trend, we also included fixed terms of whole colony size and whole colony trend. We centred and scaled all continuous variables for each sub‐colony to standardize size and trend measures at both the sub‐colony and whole colony levels. The measure of site quality was the average breeding success for a site in all years except the current year, to ensure that this value was independent of the response variables within regression modelling. All models were fitted with a binomial error structure and a ‘logit’ link. All proportional measures were weighted by the number of observations to account for any differences in the number of records for each site.

Hypothesis 1 The relationship between site occupancy and sub‐colony size and trend.

We used GLMMs to test whether higher quality sites were more likely to be occupied than lower quality sites (Hypothesis 1a). We then tested whether the effect of site quality was greater at lower population sizes (Hypothesis 1b). Density‐dependent effects on site occupancy may be affected by population trend resulting from variation in recruitment rates of naïve individuals. Consequently, to determine whether this trend‐related effect was influencing site occupancy in our system, we tested whether the predicted negative relationship between site quality and sub‐colony size on site occupancy was intensified under more positive sub‐colony trends, when the proportion of naïve individuals was likely to be higher (Hypothesis 1c).

The response was the occupancy status of a site defined as a binary value of ‘occupied’ or ‘unoccupied’. The explanatory variables were site quality, sub‐colony size, sub‐colony trend, whole colony size and whole colony trend. To test Hypothesis 1c, we included a three‐way interaction of quality, sub‐colony size and trend. All two‐way interactions between sub‐colony measures were also included. The model included a nested random effect of site within sub‐colony.

Hypothesis 2 The influence of sub‐colony size and trend on average quality of occupied sites.

We used the same model structure to test whether the average quality of occupied sites was higher at lower sub‐colony sizes (Hypothesis 2a), and whether the relationship between average quality and sub‐colony size varied with trend (Hypothesis 2b). The response was the average quality of the sites that were occupied in each sub‐colony in each year. We modelled this response as a binomial proportion of the total number of successful breeding events at a site/ total number of breeding attempts at that site. We recalculated this value for each year of data. However, we excluded the current year’s data from this measure to ensure that the quality of a site was not dependent on the success at the site in the current year. The explanatory variables were sub‐colony size, sub‐colony trend, whole colony size and whole colony trend. To test whether the average quality of occupied sites was higher under more positive sub‐colony trends (Hypothesis 2b), we included a two‐way interaction of sub‐colony size and trend.

Hypothesis 3 The relationship between average breeding success and sub‐colony size and trend.

We used the same model structure to test whether average breeding success was higher at lower sub‐colony sizes (Hypothesis 3a), and whether this effect varied with sub‐colony trend (Hypothesis 3b). The response was the average breeding success of sites in each sub‐colony in each year. This response was modelled as a binomial proportion of the number of successful breeding attempts/the total number of attempts in a year. The same explanatory and random variables were used as in the analyses of average quality. To test whether this relationship then varied with sub‐colony trend (Hypothesis 3b), we included a two‐way interaction of sub‐colony size and trend.

Hypothesis 4 The relationship between the proportion of new vs reoccupied sites across sub‐colony trend phases.

We used three generalized linear mixed‐effects models to quantify how sub‐colony size and sub‐colony trend phase affected the proportion and quality of new and reoccupied sites. Sub‐colony trend phase was a three‐level categorical variable: ‘increase’, ‘decline’ and ‘recovery’. These models allowed us to examine whether the proportion of new sites was higher in positive sub‐colony trend phases (Hypothesis 4a). The response was the proportion of new sites in each sub‐colony in each year. The explanatory variables were sub‐colony size, sub‐colony trend phase, whole colony size and whole colony trend phase. To test whether the proportion of new sites established was lower under declining sub‐colony trend phases, we also included a two‐way interaction between sub‐colony size and sub‐colony trend phase.

Next we tested whether there was a lower proportion of new sites compared to reoccupied sites in declining phases than in increasing or recovery phases (Hypothesis 4b). The response variable was the proportion of sites that were new or reoccupied in each sub‐colony in each year. The explanatory variables were occupation type (whether proportions were for new or reoccupied sites as a two‐level categorical), sub‐colony trend phase and whole colony trend phase. To determine the direction of effects among the categorical marginal effect combinations in the two‐level interaction, we extracted the least‐square means and confidence intervals for these combinations using the r package ‘lsmeans’ (Lenth, 2016).

Lastly, we tested whether new sites were of lower quality than reoccupied sites, and whether this changed with sub‐colony trend phase (Hypothesis 4c). The response was the average quality of sites for each sub‐colony in each year for new and reoccupied sites as modelled in Hypothesis 2. The explanatory variables were occupation type (new vs reoccupied), sub‐colony trend phase and whole colony trend phase. To test whether the quality of new sites was lower than reoccupied sites in all sub‐colony trend phases, we included a two‐way interaction of occupation type and sub‐colony trend phase. We used ‘lsmeans’ to extract the least‐square means for each level of the marginal effect.

### 2.6 Model validation

We used the r package ‘lme4’ (Bates et al., 2001) to run all mixed models. We ran models for all potential combinations of the fixed effects, including the inclusion/exclusion of whole terms and interactions. To compare model fit, we used Akaike’s information criterion and a nested approach, such that we selected the model with the lowest AIC (where ΔAIC > 2) (Burnham & Anderson, 1998), and where model ΔAIC < 2, we selected the simplest model (Arnold, 2010). We considered terms to have a clear effect on the response variables if their confidence intervals did not include zero (Zuur et al., 2009). For all other tests, we used a significance threshold of *p* < 0.05.

We checked that collinearity between fixed and between random effects did not exceed 0.7, because this could lead to the results of the models being distorted (Dormann et al., 2013). We inspected residual plots of fixed effects to ensure they were randomly distributed. All continuous variables were modelled as mean‐centred and scaled.

All means are presented with associated standard errors, except when stated otherwise. All statistical analyses were carried out in R version 3.6.1(R Core Team, 2019). Model prediction values were extracted using the package ‘sjPlot’ (Lüdecke, 2019). All graphs presented were produced using the r packages ‘ggplot2’ (Wickham, 2016, p. 2) and ‘ggpubr’ (Kassambara, 2019), except where indicated otherwise.

## 3 RESULTS

### 3.1 Sub‐colony size and trend

**FIGURE 2:**
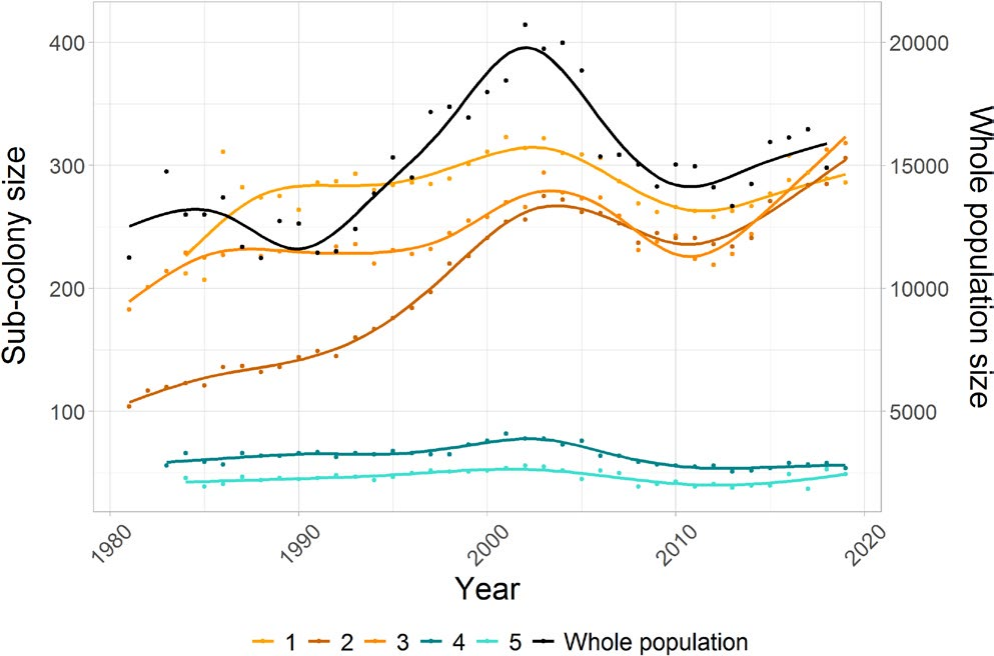
The number of breeding pairs in the five study sub‐colonies and the whole colony from 1981 to 2018

The number of breeding pairs in sub‐colonies increased from 1981 until 2002, then declined until 2013, after which numbers increased again until 2018 (Figure 2). We identified between three and six break points in the time series depending on the sub‐colony (Supplementary 2). The sequence and timing of phases and overall trend was consistent between sub‐colonies with all sub‐colonies including a sequence of increase, decline and recovery (Figure 2). Trend phases were also consistent between sub‐colonies and the whole colony apart from an initial minor declining phase in the whole colony that was not observed in sub‐colonies.

### 3.2 Relationship between site occupancy and sub‐colony size and trend

The best‐supported model included all sub‐colony‐level fixed effects (site quality, sub‐colony size and sub‐colony trend) and the three‐way interaction between these effects. Four models received some support, with a ∆AIC within 1.04, all of which included this three‐way interaction. The ∆AIC of the fifth best performing model was 51.56, hence all other models received essentially no support (detailed model outputs in Supplementary 3). These top four models differed in their inclusion of the terms whole colony size and whole colony trend. However, neither of the two whole colony terms had a significant effect in any of these models (full table of effects in Supplementary 3).

**FIGURE 3:**
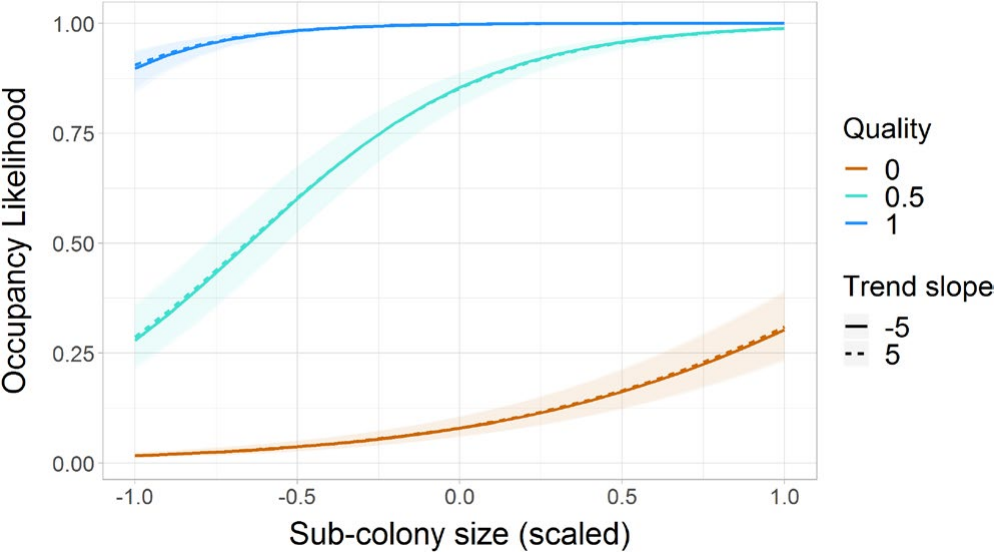
GLMM predictions (mean ± 95% CI) of the probability of a breeding site being occupied in relation to sub‐colony size (scaled and mean‐centred) and site quality. Probabilities are presented for three predicted site quality values: 1 (dark blue), 0.5 (light blue) and 0 (orange)

The final model explained 66% of the variation in the data (Table 2). Sites of higher quality were more likely to be occupied and bred at than lower quality sites at all population sizes (estimate = 8.01, 95% CI = 7.83, 8.21; Table 2; Figure 3). This evidence supports Hypothesis 1a that guillemots are able to discern site quality and are more likely to occupy the highest quality sites.

**TABLE 2:** Output from linear mixed‐effects model assessing the effect of sub‐colony size, trend and site quality on site occupancy. Significant fixed terms are shown in bold

	Estimate	Standard error	95% Confidence interval
**Fixed effects**			
**Intercept**	**−2.39**	**0.15**	**−2.68, −2.06**
**Sub‐colony size**	**1.77**	**0.08**	**1.61, 1.94**
**Quality**	**8.01**	**0.09**	**7.83, 8.21**
**Sub‐colony trend**	**0.03**	**0.01**	**0.02, 0.04**
**Sub‐colony size × quality**	**0.89**	**0.18**	**0.53, 1.29**
**Sub‐colony size × sub‐colony trend**	**0.03**	**0.01**	**0.01, 0.06**
**Sub‐colony trend × quality**	**−0.13**	**0.01**	**−0.16, −0.11**
**Sub‐colony size × quality × sub‐colony trend**	**−0.26**	**0.03**	**−0.34, −0.2**
**Random effect variances**			
Site ID	0.57		
Colony	0.69		
Year	0.01		

*Note*: Marginal *R* ^2^ = 0.55, Conditional *R* ^2^ = 0.66, Number of observations = 59,009.

Contrary to Hypothesis 1b, the effect of site quality was lower at lower population sizes (sub‐colony size × quality: 0.89, 95% CI = 0.53, 1.29). However, the positive effects of sub‐colony size and site quality meant that high‐quality sites were disproportionately used at lower population sizes (Figure 3). The effect of sub‐colony trend on occupancy itself was minimal (sub‐colony trend = 0.03, 95% CI = 0.02–0.04; Table 2). In support of Hypothesis 1c, the occupation of higher quality sites at lower sub‐colony sizes was modulated by sub‐colony trend such that the probability of occupancy increased under more positive trends (quality × sub‐colony size × sub‐colony trend: −0.26, 95% CI = −0.34, −0.2, see Table 2). However, the effect of this three‐way interaction was not easily discernible when plotted and so is not shown in Figure 3.

### 3.3 Relationship between the average quality of occupied sites and sub‐colony size and trend

**FIGURE 4:**
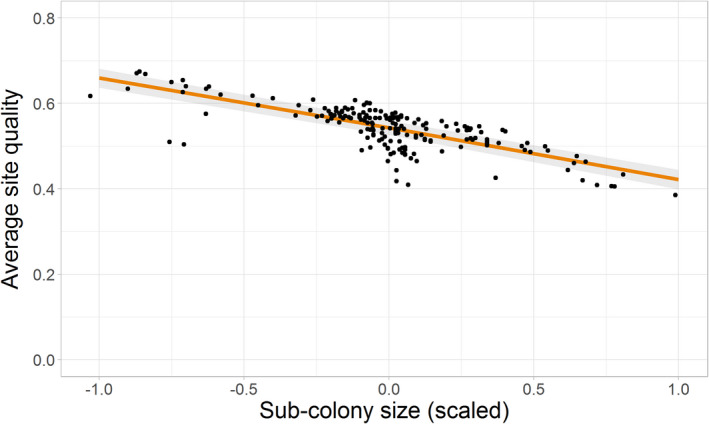
Average quality of occupied sites in each year in relation to sub‐colony size (mean‐centred and scaled). Raw data (points) and GLMM predictions (fitted line ± 95% CI) shown

As sub‐colony size increased, average site quality decreased, providing evidence to support Hypothesis 2a (estimate = −0.54, 95% CI = −0.61, −0.47; Table 3; Figure 4). Sub‐colony trend had a significant but comparatively much smaller negative effect on average site quality (estimate = −0.008, 95% CI = −0.01, −0.003). Whole colony trend had a similarly minor, but positive, effect on average site quality (estimate = 0.006, 95% CI = 0.001, 0.01). The interactions between sub‐colony trend and sub‐colony size and the effects of whole colony trend were not retained in the best‐supported model which explained 78% of the variation in the data (Table 3 and Supplementary 4). We therefore found limited evidence to support Hypothesis 2b.

**TABLE 3:** Output from GLM assessing the effect of sub‐colony size on the average quality of occupied sites. Significant fixed terms are shown in bold

	Estimate	Standard error	95% Confidence interval
**Fixed effects**			
**Intercept**	**0.17**	**0.03**	**0.08, 0.25**
**Sub‐colony size**	**−0.54**	**0.04**	**−0.61, −0.47**
**Sub‐colony trend**	**−0.008**	**0.002**	**−0.01, −0.003**
**Whole colony trend**	**0.006**	**0.002**	**0.001, 0.01**
**Random effect variances**			
Colony	0.01		
Year	0.18		

*Note*: Marginal *R* ^2^ = 0.67, Conditional *R* ^2^ = 0.78, Number of observations = 187.

### 3.4 Relationship between average breeding success and sub‐colony size and trend

**FIGURE 5:**
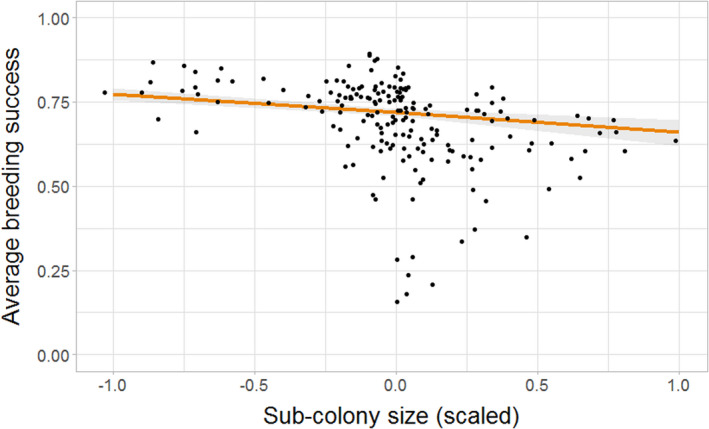
Average breeding success in each year against sub‐colony size (mean‐centred and scaled). Raw data (points) and GLMM model predictions (fitted line ± 95% CI) shown

As sub‐colony size increased, the average breeding success in a sub‐colony decreased (estimate = −0.20, 95% CI = −0.32, −0.08), supporting Hypothesis 3a (Table 4; Figure 5). Whole colony size similarly showed a negative relationship with breeding success, though the effect was much smaller (estimate = −0.01, 95% CI = −0.02, −0.005). Further, when the whole colony was increasing, breeding success increased (estimate = 0.03, 95% CI = 0.008, 0.06). Sub‐colony trend was not retained in the best‐supported model which explained 84% of the variation in the data (Table 4), though there was some limited support for the inclusion of this effect as it was retained in the second best‐supported model (∆AIC = 0.9). We therefore found limited evidence to support Hypothesis 3b.

**TABLE 4:** Output from GLM assessing the effect of sub‐colony size on the average breeding success of occupied sites. Significant fixed terms are shown in bold

	Estimate	Standard error	95% Confidence interval
**Fixed effects**			
**Intercept**	**0.83**	**0.09**	**0.64, 1.01**
**Sub‐colony size**	**−0.20**	**0.06**	**−0.32, −0.08**
**Whole colony size**	**−0.01**	**0.003**	**−0.02, −0.005**
**Whole colony trend**	**0.03**	**0.01**	**0.008, 0.06**
**Random effect variances**			
Colony	0.01		
Year	0.18		

*Note*: Marginal *R* ^2^ = 0.31, Conditional *R* ^2^ = 0.91, Number of observations = 187.

### 3.5 Relationship between the proportion of sites that were new and sub‐colony size across sub‐colony trend phases

In declining phases, the proportion of sites that were new was significantly lower than during increasing or recovering sub‐colony trend phases (estimate = −0.49 95% CI = −0.77, −0.19; Table 5). However, there was no difference in the proportion of new sites between increasing and recovery sub‐colony trend phases (estimate = 0.12, 95% CI = −0.23, 0.48; Table 5). Trend phase was the only fixed effect retained in the best‐supported model (Supplementary 6; 60% of variation explained). Together, these results show support for Hypothesis 4a.

**TABLE 5:** Output from GLMM testing the relationship between the proportion of sites established that were new, sub‐colony trend phase and whole colony trend phase. The trend phase of ‘increase’ was used as a reference level. Significant fixed terms are shown in bold

	Estimate	Standard error	95% Confidence interval
**Fixed effects**			
**Intercept**	**−3.48**	**0.18**	**−3.89, −3.07**
**Sub‐colony trend phase**			
**Decline**	**−0.49**	**0.15**	**−0.77, −0.19**
Recovery	0.12	0.17	−0.23, 0.48
**Random effect variances**			
Colony	0.12		
Year	0.13		

*Note*: Marginal *R* ^2^ = 0.14, Conditional *R* ^2^ = 0.60, Number of observations = 145.

**FIGURE 6:**
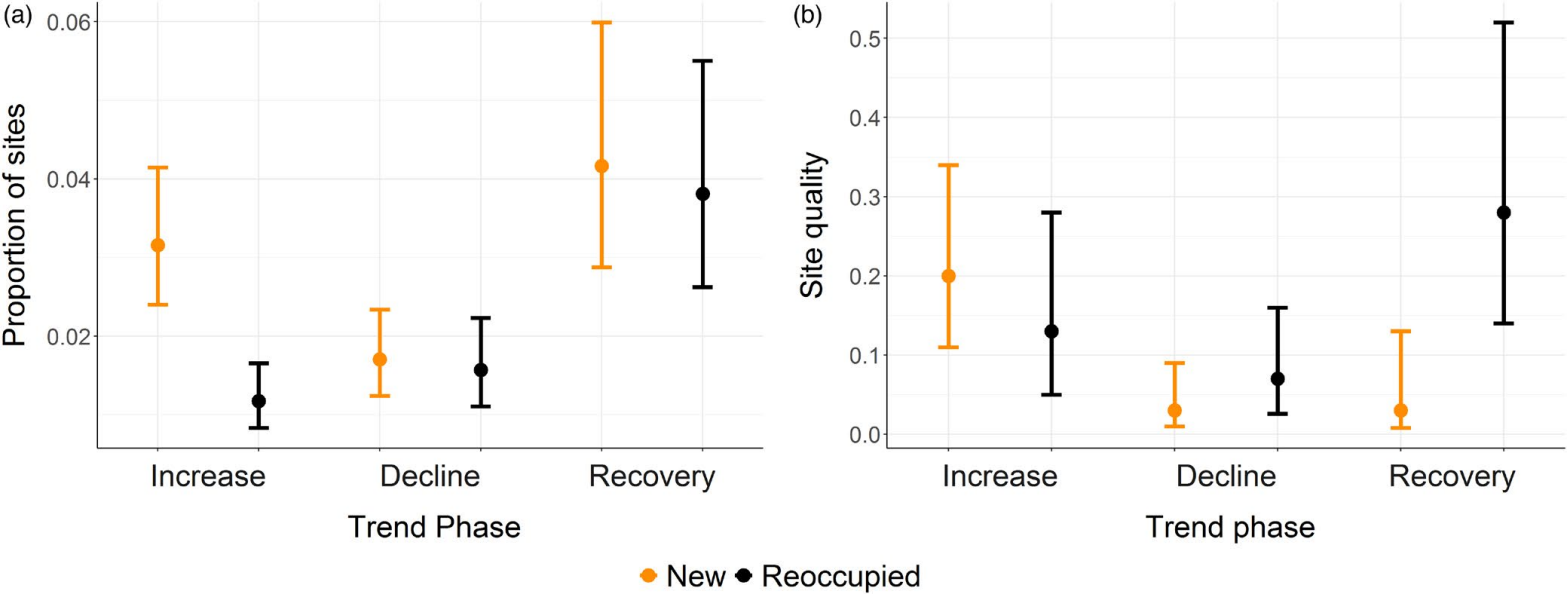
(a) The proportion, and (b) the quality of new and reoccupied sites occupied in three sub‐colony trend phases, presented as the mean (circle) ± 95% CI (whiskers) proportion for each year of a phase

The fixed effects of occupation type (whether a site was ‘new’ or ‘reoccupied’) and sub‐colony trend phase (increase, decline or recovery), and the two‐way interaction between these were retained in the best‐supported model testing the effects on the proportion of sites occupied. Whole colony trend phase was retained in the second best‐supported model indicating limited support for the inclusion of this effect (∆AIC = 2.84) (Supplementary 7; 74% variation explained by best‐supported model). During the initial increase phase, the proportion of new sites established was higher than the proportion that was reoccupied (least‐square means; new: 0.03, 95% CI = 0.02, 0.05; reoccupied: 0.01, 95% CI = 0.007, 0.02, Table 6; Figure 6a). However, there was no equivalent difference in the declining or recovering phases (least‐square means; declining: new: 0.017, 95% CI = 0.011, 0.026; reoccupied: 0.016, 95% CI = 0.01, 0.03; recovering: new: 0.042, 95% CI = 0.025, 0.068; reoccupied: 0.038, 95% CI = 0.02, 0.06). We therefore found no support for Hypothesis 4b.

**TABLE 6:** Output from GLMM of the proportion of sites established in different trend phases that were either ‘new’ or ‘reoccupied’. The trend phase of ‘increase’, and the occupation type of ‘new’ were used as reference levels. Significant fixed terms are shown in bold

	Estimate	Standard error	Confidence interval
**Fixed effects**			
**Intercept**	**−3.42**	**0.14**	**−3.72, −3.14**
**Site type**	**−1.01**	**0.12**	**−1.26, −0.77**
**Sub‐colony trend phase**			
**Decline**	**−0.63**	**0.17**	**−0.96, −0.30**
Recovery	0.28	0.22	−0.13, 0.74
**Occupation type × phase**			
**Decline**	**0.93**	**0.18**	**0.57, 1.28**
**Recovery**	**0.92**	**0.16**	**0.61, 1.23**
**Random effect variances**			
Colony	0.02		
Year	0.28		

*Note*: Marginal *R* ^2^ = 0.31, Conditional *R* ^2^ = 0.74, Number of observations = 245.

The fixed effects of occupation type and sub‐colony trend phase, the two‐way interaction between these and whole colony trend phase were retained in the best‐supported model testing the effects on the quality of sites (Supplementary 8; 86% of variation explained). No other models received reasonable support in the data, since ∆AIC to the next best‐supported model was 6.85. There was no overall difference between the quality of new and reoccupied sites (estimate = −0.54, 95% CI = −1.17, 0.03; Table 7). However, new sites in the recovery phase were of lower quality than reoccupied sites (least‐square means; new: 0.03, 95% CI = 0.008, 0.13; reoccupied: 0.28, 95% CI = 0.14, 0.51; Figure 6b). There were no differences between the quality of new and reoccupied sites in other phases (least‐square means; increase: new: 0.2, 95% CI = 0.11, 0.34; reoccupied: 0.13, 95% CI = 0.05, 0.28; decline: new: 0.03, 95% CI = 0.01, 0.09; reoccupied: 0.07, 95% CI = 0.03, 0.16). The retention of whole colony trend phase in the model indicated that processes acting at a wider level may be affecting the quality of reoccupied and newly established sites such that their quality is higher in negative trend phases when numbers are declining. Therefore, we found limited support for Hypothesis 4c.

**TABLE 7:** Output from GLMM of the quality of sites established in different trend phases that were either ‘new’ or ‘reoccupied’. The trend phase of ‘increase’ and the occupation type of ‘new’ were used as reference levels. Significant fixed terms are shown in bold

	Estimate	Standard error	Confidence interval
**Fixed effects**			
**Intercept**	**−1.44**	**0.21**	**−1.95, −1.02**
Occupation type	−0.54	0.31	−1.17, 0.03
**Sub‐colony trend phase**			
**Decline**	**−2.01**	**0.36**	**−2.78, −1.34**
**Recovery**	**−1.97**	**0.64**	**−3.34, −1.34**
**Whole colony trend phase**			
**Decline**	**0.71**	**0.20**	**0.31, 1.10**
Recovery	−0.53	0.51	−1.52, 0.50
**Occupation type × sub‐colony trend phase**			
**Decline**	**1.30**	**0.53**	**0.27, 2.37**
**Recovery**	**2.95**	**0.58**	**1.90, 4.18**
**Random effect variances**			
Colony	0.09		
Year	0.001		

*Note*: Marginal *R* ^2^ = 0.18, Conditional *R* ^2^ = 0.22, Number of observations = 245.

## 4 DISCUSSION

In this study, we explored the role of the buffer effect in population regulation, where high‐quality breeding sites are a potentially limiting resource. In colonially breeding guillemots, we found that individuals bred disproportionately at the highest quality sites at smaller sub‐colony sizes resulting in higher average site quality and breeding success at lower population sizes. In further support of site‐dependent regulation, we found that average site quality and breeding success varied little with sub‐colony trend. However, when the population was recovering after a decline, new sites and vacant previously occupied sites were occupied at similar frequencies despite the latter being of higher quality. Together, our findings indicate that there may be some limit to the capacity of site‐dependent regulation to provide resilience in recovering populations.

A higher occupation rate of higher quality sites has been widely found in past studies in a variety of species (e.g. Morris & MacEachern, 2010; Rodenhouse et al., 1997). There is also evidence for this pattern of occupation and consequent population regulation in both increasing and decreasing populations (Gill et al., 2001; Sullivan et al., 2015). We demonstrate that population trend had only a very minor effect on the probability of a site being occupied; the effect of trend was >50× smaller than that of the individual and combined effects of site quality and sub‐colony size. In addition, we found only a minor effect of trend on average site quality. To our knowledge, this is the first study to demonstrate consistent patterns of occupancy in relation to breeding site quality and sub‐colony size in a wild population. We found a minor effect of whole colony trend on average site quality, indicating that wider extrinsic factors affecting the whole colony, such as prey availability, may also be having some influence on the patterns of site occupation across the population. When extrinsic conditions are more favourable, individuals across the colony as a whole may be in better condition prior to and during breeding. This feature of colonial species contrasts with the situation in territorial species where extrinsic factors may operate on a more local scale resulting in varying impacts on breeders with potential consequences for site‐dependent variation (Hinks et al., 2015).

The transmission of public information on site quality is important for decisions about the occupation of breeding sites (Doligez et al., 2002). Thus, a lack of evidence for a large effect of sub‐colony trend on site occupancy may indicate that the relative proportions of naïve individuals may covary with the proportion of more experienced individuals across sub‐colony trends. The recruitment rate of these individuals may be governed by wider whole colony processes, which could explain the effect of whole colony trend on the average quality of occupied sites. Any increase in numbers of these less well‐informed individuals (Dittmann & Becker, 2003) appears then to be counteracted by a similar relative change in number of more experienced individuals potentially making more informed decisions about site occupation. In support of this, an earlier study on this population found that recruitment of individuals to the breeding population decreased linearly with increasing population size, suggestive of a density‐dependent mechanism of naïve birds entering the population (Crespin et al., 2006). Furthermore, in many long‐lived species, first‐time breeders are not entirely naïve, because they may take several years to recruit into the breeding population during which time they accumulate knowledge of potential breeding sites (Brown & Rannala, 1995; Schjørring et al., 1999). This is the case in guillemots with individuals typically attending the colony for several years prior to their first breeding attempt potentially enabling them to become familiar with the physical qualities of sites and their recent breeding success (Halley et al., 1995). The physical characteristics of most guillemot breeding sites on the Isle of May remained constant over the study period (as has also been found in other colonies: Birkhead & Nettleship, 1987). Thus, information gathered by prospecting individuals should be a reliable cue for the future quality of a site. However, it is important to note that the value of social information available to individuals may vary with local population size, that is public information is less informative when there are fewer breeding attempts to infer site quality from (Doligez et al., 2003). Overall, the prospecting behaviour of immatures prior to breeding may also act to minimize any negative effects of a negative trend on site occupancy, as few individuals will be completely naïve about the physical characteristics and breeding success at sites. Importantly, the consistently higher probability of occupation of the highest quality sites demonstrates that site‐dependent regulation provides resilience to populations across population sizes and trends through maximizing potential fitness via the occupation of higher quality sites.

A key mechanism whereby site‐dependent regulation may operate in a consistent manner across all trends is through individuals reoccupying vacant historically used sites as opposed to establishing new sites of lower quality (Espie et al., 2004). We expected individuals to successfully discern the quality of sites and reoccupy those previously used since they are of higher average quality than new sites, potentially through acquiring public information (Doligez et al., 2002). In agreement with this, we found that the proportion of new sites was lower in the declining phase. Furthermore, a greater proportion of new sites was occupied in positive sub‐colony trend phases, which contributed to the decline in average breeding success we found at larger sub‐colony sizes. Despite the higher quality of reoccupied sites, we found no difference between the proportion of new and reoccupied sites during the recovery phase. This finding suggests that the average quality of sites in increasing and recovering populations may perhaps be limited by the opportunity or ability of first‐time breeders, which may represent a substantial proportion of the population, to discern site quality accurately because they have had limited access to public information. The positive effect of whole colony trend phase on the quality of new and reoccupied sites indicates that there may be additional extrinsic factors, such as prey availability, that are affecting the quality of occupied sites when the population is recovering. Moreover, for first‐time breeders, the reliability of breeding success information at sites will improve over time, but for new sites they will only have information on physical characteristics initially. As it is likely that individuals use a combination of physical characteristics and breeding outcomes to make decisions on where to breed, we would expect less experienced birds to make less optimal decisions because they have less complete information on historical breeding success at sites. Following this, the acquisition of public information has been clearly linked to the local density of breeding pairs; individuals are more likely to accurately discern site quality when density is higher (Doligez et al., 2003; Forsman et al., 2008). In addition, as colonially breeding species are more likely to breed close to conspecifics (Stamps, 1994), first‐time breeders may be limited in where they can breed by the presence of established breeders. Consequently, while this public knowledge is re‐acquired by more experienced breeders that have survived the period of decline or acquired by first‐time breeders, those smaller populations may be at increased vulnerability and likelihood of declines in the early stages of recovery. Further work involving detailed behavioural observations of individuals is needed to establish if prospecting behaviour and site choice differs under different population conditions.

In keeping with our findings on the effect of sub‐colony size on site occupancy and average site quality, average breeding success increased at lower sub‐colony sizes, and these effects were broadly consistent across sub‐colony trends. This outcome is in agreement with previous studies that found links between breeding habitat quality and population fitness (Espie et al., 2004; Kokko et al., 2004). Thus, while the buffer effect will not be the only mechanism driving the relationship between productivity and sub‐colony size, the quality of sites available to individuals will contribute to the likelihood of having a successful breeding attempt and offer resilience to populations experiencing declines caused by external factors. However, breeding success may be indirectly affected by a large number of highly variable year‐specific drivers such as the availability of prey (Lewis et al., 2009; Wanless et al., 2005) and other forms of environmental stochasticity (Ambrosini et al., 2006) that may affect the population average breeding success, as demonstrated by the relatively large confidence intervals of this effect in our study. The importance of extrinsic factors is supported by the negative effect of whole colony size and the positive effect of trend indicating that additional factors affecting the whole population are contributing to average breeding success. A factor that may intensify this effect is that at smaller relative population sizes those individuals that have survived overwinter and are in a good enough condition to breed are more likely to be those individuals of highest quality (Espie et al., 2004; Robert et al., 2012). This may result in the ‘best’ individuals breeding at the ‘best’ sites, leading to a higher average productivity for the population. Distinguishing between the inherent quality of individuals and the quality of their territories is challenging (Bergeron et al., 2011; Germain & Arcese, 2014). We were unable to do this in our study because turnover of individuals is low (86% of breeding attempts by individuals took place at the same site from the previous year over the course of this study). However, as found in previous studies that have been able to disentangle site and individual quality effects (Espie et al., 2004; Newton, 1991), it is likely that both effects are present in our study system. In particular, it is known that the physical properties of sites directly influence breeding success in our study population (Harris et al., 1997), so we are confident that a true effect of site quality is present, but the degree to which this effect is modulated by intrinsic individual quality remains unknown. Testing the relative importance of these two effects could be achieved through the careful use of an experimental approach to manipulate nest sites, such as through the use of artificial breeding sites in a guillemot colony in the Baltic (Hentati‐Sundberg et al., 2012).

Overall, we present important new evidence that site‐dependent regulation, whereby the most productive sites are disproportionately occupied at lower population sizes leading to improved breeding success, may be a key mechanism providing resilience in populations irrespective of population status. However, the occupancy of new sites even when previously occupied sites of higher quality are available suggests that the recovery of populations may be somewhat hampered by the inability of individuals to accurately assess site quality. There is a need for future studies across a wide range of species, life histories and habitats to assess how individuals discern site quality, including the importance of information transfer between conspecifics, and to distinguish between site and individual quality effects. Such advances would greatly enhance our understanding of the precise mechanisms underpinning the potential for site‐dependent regulation to provide resilience to threatened populations.

## CONFLICT OF INTEREST

The authors declare no competing interests.

## AUTHORS' CONTRIBUTIONS

S.B., F.D., S.W. and M.P.H. conceived the ideas and designed the experimental methodology; M.P.H., S.W., M.A.N. and S.B. collected the data; S.B. analysed the data, supported by K.S.; S.B. led the writing of the manuscript supported by F.D., S.W., M.P.H., K.S. and J.G. All authors contributed critically to the drafts and gave final approval for publication.

## Supporting information

Supinfo

## Data Availability

The data used in this study are available from the Environmental Information Data Centre 10.5285/33b42f0a‐12a5‐47fe‐aaaf‐25f4ee5e13a5 (Bennett et al., 2022).
