# Supplementary material for: Site‐dependent regulation of breeding success: Evidence for the buffer effect in the common guillemot, a colonially breeding seabird
Source: J Anim Ecol. 2022 Feb 25;91(4):752–65. doi: 10.1111/1365-2656.13674 (PMC9305850; doi:10.1111/1365-2656.13674)
Supplement: Supplementary file 1 — Supinfo [file JANE-91-752-s001.docx]

**Supplementary material:**

**S.1 The relationship between the physical characteristics of a site and breeding success:**

**Methods:**

Previous analyses used the average breeding success at a site and its physical characteristics as alternative measures of site quality (Harris et al (1997); Kokko et al (2004)). These studies have shown that six physical attributes of a site are highly correlated with breeding success - number of neighbours, the number of walls, the site’s distance from the top of the cliff, the floor, site type (see below for definitions), and site slope. Accordingly, we performed a principle-component analysis (PCA) to obtain a composite measure of a site’s physical quality, as several of the terms were significantly correlated with each other. The PCA analysis included four of these six physical characteristic measures of sites. We did not include the site type in our analysis to reduce the risk of over-fitting due to other physical characteristics contributing to this measure for two out of four site types: sites on ‘niches’ were accounted for through the number of neighbours that a site has (n sites with this site type= 93/1230), and sites on ‘platforms’ were based on two other physical characteristics, those with no walls and with no neighbours (n=113/1230). Of the remaining 1020 sites, 957 were categorised as a ‘ledge’ and 63 as ‘on stones’. To test whether site type was still related to breeding success as found in Harris et al (1997), we ran a linear regression between site type and average breeding success for sites on ledges or stones and found no significant relationship (estimate=0.19, se=0.26, p=0.45). Hence we did not include site type in our analysis. We also excluded floor type of a site since in some cases a site changed category through accumulation or loss of soil or guano. The resulting four variables retained in the PCA analysis are presented in Table S1.1.

**Table S1.1 Physical characteristics of guillemot breeding sites in the Principal Component Analysis**

| **Physical site characteristic** | **Description** | **How measured?** |
| --- | --- | --- |
| *Number of neighbours* | The number of neighbouring sites sharing a boundary in the year that a site was established | Counted from annotated photographs |
| *Number of walls* | The number of sides surrounding a site that were vertical rock | Counted from annotated photographs or visually in-situ |
| *Slope of site* | The angle of elevation given to the nearest 5 degrees | In situ using a spirit level |
| *Distance from top of cliff* | The distance of a site from the top of the cliff in metres | Measured from the top of the cliff using a rope marked at 1m intervals |

We retained components with an eigenvalue of >1, comprising two of the four estimated components explaining 65% of the variance. To test whether the breeding success of a site was related to its physical characteristics we ran a mixed model with a site’s average breeding success as the response variables and with the top two principle components, and an interaction between them, as the explanatory variables. We included sub-colony as a random effect as the colonies varied markedly in their physical structure, the number of occupied sites and aspect.

**Results:**

The two principal components that together explained 65% of variance are shown in Fig.S1.1 and Table.S1.2.


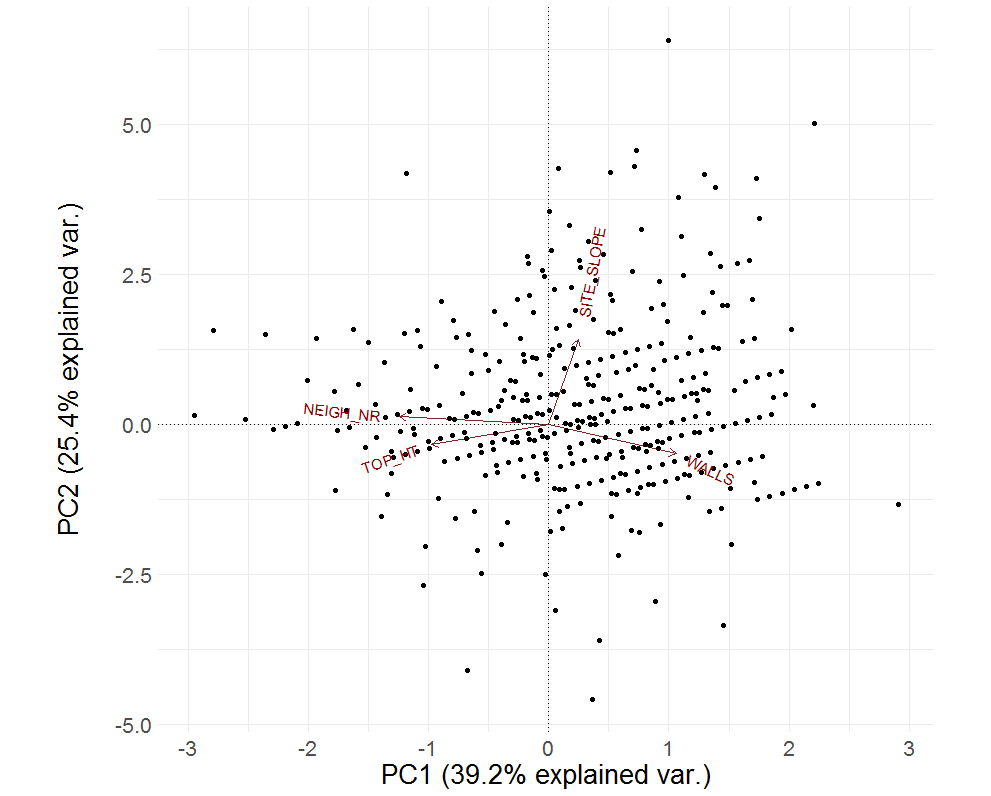


**Figure S1.1 Principle components analysis biplot explaining variance in four physical characteristics of breeding sites. Arrows indicate the direction of increasing values for each of the variables. Plot produced using ‘ggbiplot’ (Vincent, 2011).**

**Table S1.2 The importance and relative contributions of principle components explaining variation in physical breeding site characteristics**

| *Importance of components* | PC1 | PC2 | PC3 | PC4 |
| --- | --- | --- | --- | --- |
| *Eigenvalue* | 1.57 | 1.02 | 0.83 | 0.58 |
| *Standard deviation* | 1.25 | 1.00 | 0.91 | 0.76 |
| *Proportion of variance* | 0.39 | 0.25 | 0.21 | 0.15 |
| *Cumulative proportion* | 0.39 | 0.65 | 0.85 | 1.00 |
| *Number of observations: 1215* | |  |  |  |

A GLM showed that there was a positive relationship between these two components and the mean breeding success of a site (see Table S1.3). So, we are confident that the measure of average breeding success is an appropriate measure of site quality.

**Table S1.3 Results from a general linear model assessing the relationship between two principle components of physical characteristics and the mean breeding success of a site**

|  | Estimate | Standard error | 95% Confidence interval |
| --- | --- | --- | --- |
| ***Intercept*** | **0.61** | **0.008** | **0.59, 0.62** |
| ***PC1*** | **0.03** | **0.006** | **0.02, 0.04** |
| ***PC2*** | **0.04** | **0.008** | **0.02, 0.05** |
| ***PC1 * PC2*** | **-0.01** | **0.006** | **-0.02, 0.004** |
|  |  |  |  |
| **Number of observations: 1213** | | |  |

Further these physical characteristics have remained little changed across our study period. Site slopes are unlikely to have changed because there has been limited accumulation of guano or soil on the rock surface. The number of walls and the distance from the top of the cliff are also little changed as there has been no substantial rock fall altering the structure of sub-colonies (in contrast to that observed in other colonies (Heubeck et al., 2014)).

By contrast, the fourth measure of physical site characteristics that we assess, the number of neighbours, will have changed for some sites. However, this variable was highly correlated with distance from the top of the cliff (Figure S1.1), so we do not think a change in the number of neighbours will have significantly affected the relationship we identified between physical characteristics and average breeding success.

Accordingly, given the stability of physical characteristics of guillemot breeding sites at this colony over time, we are confident that the quality of sites will have been consistent throughout the study period, as also found by Birkhead & Nettleship (1987)**.**

**S.2 Identification of break points in population trend:**

**Table S2.1 Significant changes in the trend of population size, termed trend phases, identified for each sub-colony. The first and last years of trend phases are the break points where the overall population trend significantly changed. The slope of change in population size was the change in population size per year during a trend phase period. The sub-colony trend was calculated as the slope of change in population size as a proportion of the average population size in each period. Trend phase describes whether the population was increasing (with a positive sub-colony trend), declining (with a negative sub-colony trend), or recovering (with a positive sub-colony trend following a decline).**

| ***Colony*** | Trend phase period | Slope of change in population size | Standard error | Sub-colony trend (%) | Trend phase |
| --- | --- | --- | --- | --- | --- |
| *Whole colony* | 1983-1988 | -596.94 | 175.1 | -4.63 | Decline |
|  | 1989-2004 | 645.83 | 59.9 | 4.16 | Increase |
|  | 2004-2013 | -620.02 | 166.3 | -3.91 | Decline |
|  | 2013-2018 | 417.49 | 250.7 | 2.75 | Recovery |
|  |  |  |  |  |  |
| *Sub-colony 1* | 1984-1990 | 9.11 | 6.71 | 3.49 | Increase |
|  | 1990-1997 | 1.48 | 1.29 | 0.57 | Increase |
|  | 1997-2001 | 9.8 | 0.82 | 3.25 | Increase |
|  | 2001-2007 | -4.89 | 1.21 | -1.58 | Decline |
|  | 2007-2013 | -3.32 | 1.29 | -1.24 | Decline |
|  | 2013-2018 | 4.64 | 1.30 | 1.65 | Recovery |
|  |  |  |  |  |  |
| *Sub-colony 2* | 1981-1991 | 3.78 | 0.43 | 2.92 | Increase |
|  | 1991-2003 | 11.81 | 0.38 | 5.67 | Increase |
|  | 2003-2013 | -3.95 | 0.61 | -1.59 | Decline |
|  | 2013-2018 | 10.37 | 2.66 | 3.69 | Recovery |
|  |  |  |  |  |  |
| *Sub-colony 3* | 1981-1993 | 3.05 | 0.73 | 1.37 | Increase |
|  | 1993-2002 | 5.15 | 0.87 | 2.11 | Increase |
|  | 2002-2010 | -6.13 | 1.70 | -2.34 | Decline |
|  | 2010-2018 | 11.42 | 2.00 | 4.31 | Recovery |
|  |  |  |  |  |  |
| *Sub-colony 4* | 1983-2001 | 0.64 | 0.15 | 1.01 | Increase |
|  | 2001-2011 | -2.51 | 0.36 | -4.74 | Decline |
|  | 2011-2018 | 1.54 | 0.32 | 0.61 | Recovery |
|  |  |  |  |  |  |
| *Sub-colony 5* | 1984-2002 | 0.67 | 0.91 | 1.41 | Increase |
|  | 2002-2007 | -1.31 | 0.82 | -2.54 | Decline |
|  | 2007-2018 | 0.2 | 0.36 | 0.46 | Recovery |

**S.3 AIC table for occupancy models:**

**Table S3.1 AIC table of generalised linear mixed-effects models with different fixed effect term structures to investigate the relationship between the likelihood of a breeding site being occupied, and the relative quality of that site, sub-colony size and trend and whole colony size and trend. Where two models were within 2 AIC units of one another we selected the model with the simplest model structure. The most supported model with the simplest model structure is shown in bold.**

| *Fixed effects structure* | Number of parameters | AIC | ∆AIC |
| --- | --- | --- | --- |
| *Site quality * Sub-colony size * Sub-colony trend + Whole colony trend* | 12 | 41561.97 | 0 |
| ***Site quality * Sub-colony size * Sub-colony trend*** | **11** | **41562.36** | **0.39** |
| *Site quality * Sub-colony size * Sub-colony trend + Whole colony size* | 12 | 41562.59 | 0.62 |
| *Site quality * Sub-colony size * Sub-colony trend + Whole colony size + Whole colony trend* | 13 | 41563.01 | 1.04 |

All other models (72/76) had ∆AIC of >51.56 (range 51.56- 3610.10) and so received little support.

**Table S3.2 Outputs of linear mixed-effects models with some support assessing the effect of sub-colony size, trend and site quality on site occupancy. Significant fixed terms are shown in bold.**

| **Model 2:** *∆AIC 0.39 to top model* | | | |
| --- | --- | --- | --- |
| ***Fixed effects:*** | Estimate | Standard error | 95% Confidence interval |
| ***Intercept*** | **-2.37** | **0.15** | **-2.68, -2.06** |
| ***Sub-colony size*** | **1.77** | **0.08** | **1.61, 1.94** |
| ***Quality*** | **8.01** | **0.09** | **7.83, 8.21** |
| ***Sub-colony trend*** | **0.03** | **0.01** | **0.02, 0.04** |
| ***Sub-colony size* quality*** | **0.91** | **0.18** | **0.53, 1.29** |
| ***Sub-colony size* sub-colony trend*** | **0.13** | **0.01** | **0.16, 0.11** |
| ***Sub-colony trend * quality*** | **-0.03** | **0.01** | **-0.01, -0.06** |
| ***Sub-colony size* quality* Sub-colony trend*** | **-0.27** | **0.03** | **-0.20, -0.34** |
|  |  |  |  |
| ***Random effect variances*** |  |  |  |
| *Site ID* | 0.58 |  |  |
| *Colony* | 0.73 |  |  |
| *Year* | 0.01 |  |  |
|  |  |  |  |
| \| *Marginal R^2^=0.55 , Conditional R^2^= 0.66, Number of observations= 59009* \| \| --- \| \|  \| | | | |
| **Model 3:** *∆AIC 0.62 to top model* | | | |
| **Fixed effects:** | **Estimate** | **Standard error** | **95% Confidence interval** |
| ***Intercept*** | **-2.51** | **0.06** | **-2.63, -2.39** |
| ***Sub-colony size*** | **1.82** | **0.07** | **1.67, 1.96** |
| ***Quality*** | **8.06** | **0.10** | **7.87, 8.25** |
| ***Sub-colony trend*** | **0.03** | **0.004** | **0.025, 0.04** |
| ***Sub-colony size* quality*** | **0.64** | **0.18** | **0.28, 1.004** |
| *Sub-colony size* sub-colony trend* | 0.02 | 0.01 | -0.0003, 0.04 |
| ***Sub-colony trend * quality*** | **-0.13** | **0.01** | **-0.15, -0.11** |
| ***Sub-colony size* quality* Sub-colony trend*** | **-0.24** | **0.03** | **-0.30, -0.18** |
| *Whole colony size* | 0.0009 | 0.0007 | -0.0004, 0.002 |
|  |  |  |  |
| ***Random effect variances*** |  |  |  |
| *Site ID* | 0.59 |  |  |
| *Colony* | 0.009 |  |  |
| *Year* | 0.001 |  |  |
|  |  |  |  |
| \| *Marginal R^2^=0.58 , Conditional R^2^= 0.64, Number of observations= 59009* \| \| --- \| | | | |
|  |  |  |  |
| **Model 4:** *∆AIC 1.04 to top model* | | | |
| **Fixed effects:** | **Estimate** | **Standard error** | **95% Confidence interval** |
| ***Intercept*** | **-2.51** | **0.06** | **-2.63, -2.39** |
| ***Sub-colony size*** | **1.82** | **0.07** | **1.67, 1.96** |
| ***Quality*** | **8.06** | **0.095** | **7.87, 8.25** |
| ***Sub-colony trend*** | **0.03** | **0.004** | **0.02, 0.04** |
| ***Sub-colony size* quality*** | **0.62** | **0.18** | **0.26, 0.99** |
| *Sub-colony size* sub-colony trend* | 0.02 | 0.01 | -0.0003, 0.04 |
| ***Sub-colony trend * quality*** | **-0.13** | **0.01** | **-0.15, -0.11** |
| ***Sub-colony size* quality* Sub-colony trend*** | **-0.24** | **0.03** | **-0.30, -0.18** |
| *Whole colony size* | 0.001 | 0.0007 | -0.001, 0.002 |
| *Whole colony trend* | 0.003 | 0.003 | -0.002, 0.008 |
|  |  |  |  |
| ***Random effect variances*** |  |  |  |
| *Site ID* | 0.59 |  |  |
| *Colony* | 0.01 |  |  |
| *Year* | 0.001 |  |  |
|  |  |  |  |
| *Marginal R^2^=0.58 , Conditional R^2^= 0.64, Number of observations= 59009* | | | |

**S.4 AIC table for average quality models:**

**Table S4.1 AIC table of generalised linear mixed-effects models with different fixed effect term structures to investigate the relationship between the average site quality in a given year, sub-colony size and trend and whole colony size and trend. Where two models were within 2 AIC units of one another we selected the model with the simplest model structure. The most supported model with the simplest model structure is shown in bold.**

| *Fixed effects structure* | Number of parameters | AIC | ∆AIC |
| --- | --- | --- | --- |
| ***Sub-colony size + Sub-colony trend + Whole colony trend*** | **6** | **981.92** | **0** |
| *Sub-colony size + Sub-colony trend + Whole colony trend + Whole colony size* | 7 | 984.08 | 2.16 |
| *Sub-colony size * Sub-colony trend + Whole colony trend* | 7 | 984.10 | 2.18 |
| *Sub-colony size + Sub-colony trend* | 5 | 984.92 | 3.00 |
| *Sub-colony size * Sub-colony trend + Whole colony trend + Whole colony size* | 8 | 986.29 | 4.36 |
| *Sub-colony size + Sub-colony trend + Whole colony size* | 6 | 986.52 | 4.60 |
| *Sub-colony size * Sub-colony trend* | 6 | 987.07 | 5.15 |
| *Sub-colony size* | 4 | 988.61 | 6.69 |
| *Sub-colony size * Sub-colony trend + Sub-colony size* | 7 | 988.69 | 6.77 |
| *Sub-colony size + Whole colony trend* | 5 | 989.92 | 7.99 |
| *Sub-colony size + Whole colony size* | 5 | 990.27 | 8.35 |
| *Sub-colony size + Whole colony size + Whole colony trend* | 6 | 991.83 | 9.91 |
| *Sub-colony trend + Whole colony size* | 5 | 1100.18 | 118.26 |
| *Whole colony size* | 4 | 1100.45 | 118.53 |
| *Sub-colony trend + Whole colony size + Whole colony trend* | 6 | 1101.90 | 120.00 |
| *Whole colony size + Whole colony trend* | 5 | 1102.57 | 120.65 |
| *Null* | 3 | 1112.92 | 131.00 |
| *Sub-colony trend* | 4 | 1113.34 | 131.42 |
| *Whole colony trend* | 4 | 1113.97 | 132.05 |
| *Sub-colony trend + Whole colony trend* | 5 | 1114.99 | 133.07 |


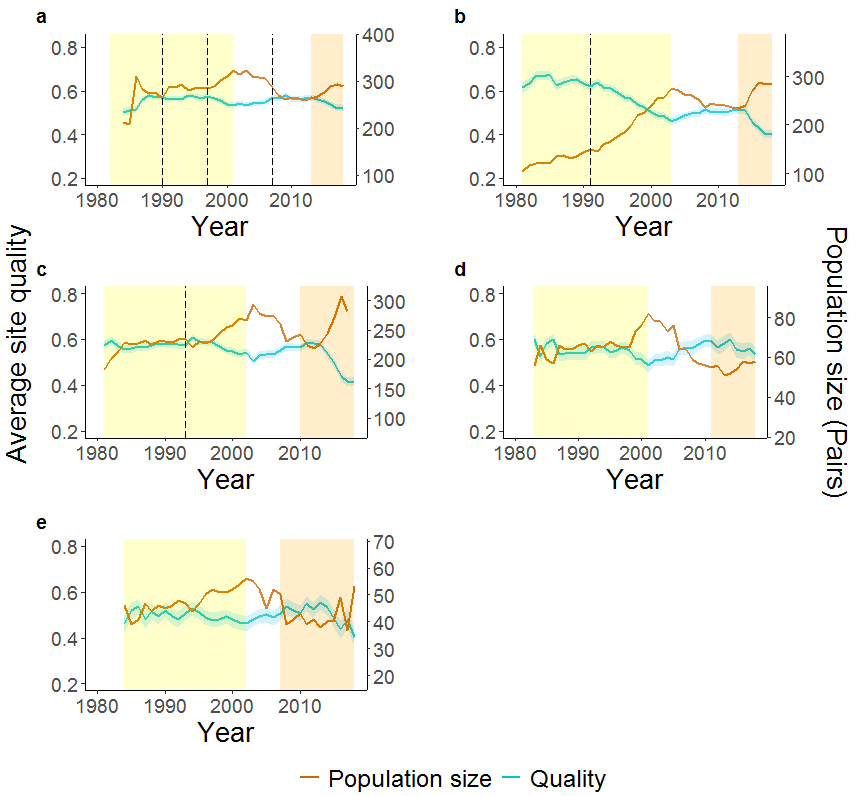
**Figure S4.1 The average quality of sites (dark orange line), population size (blue line), and population trend phase (shaded areas) over time for the five sub-colonies (a-e corresponding to sub-colonies 1-5 respectively). The standard error of the average quality is shown as orange shading. Trend phase is indicated by vertical shaded regions: increase (light orange), decline (white), and recovery (dark orange). Break points within a trend phase are indicated by vertical dashed lines. The population size axis has been rescaled to demonstrate the reciprocal relationship with average quality.**

**S.5 AIC table for average breeding success models:**

**Table S5.1 AIC table of generalised linear mixed-effects models with different fixed effect term structures to investigate the relationship between average breeding success in a given year, sub-colony size and trend and whole colony size and trend. Where two models were within 2 AIC units of one another we selected the model with the simplest model structure. The most supported model with the simplest model structure is shown in bold.**

| *Fixed effects structure* | Number of parameters | AIC | ∆AIC |
| --- | --- | --- | --- |
| ***Sub-colony size + Whole colony size + Whole colony trend*** | **6** | **1239.87** | **0** |
| *Sub-colony size + Sub-colony trend + Whole colony size + Whole colony trend* | 7 | 1240.77 | 0.90 |
| *Sub-colony size * Sub-colony trend + Whole colony size + Whole colony trend* | *8* | *1242.28* | *2.41* |
| *Sub-colony size + Whole colony size* | *5* | *1243.84* | *3.97* |
| *Sub-colony size + Sub-colony trend + Whole colony size* | *6* | *1245.20* | *5.33* |
| *Sub-colony size * Sub-colony trend + Whole colony size* | *7* | *1246.71* | *6.84* |
| *Sub-colony size* | *4* | *1248.59* | *8.71* |
| *Sub-colony size + Whole colony trend* | *5* | *1248.91* | *9.04* |
| *Whole colony size + Whole colony trend* | *5* | *1249.67* | *9.80* |
| *Sub-colony size + Sub-colony trend* | *5* | *1249.78* | *9.91* |
| *Sub-colony size + Sub-colony trend + Whole colony trend* | *6* | *1249.88* | *10.01* |
| *Sub-colony trend + Whole colony size + Whole colony trend* | *6* | *1250.71* | *10.84* |
| *Sub-colony size * Sub-colony trend* | *6* | *1251.25* | *11.38* |
| *Sub-colony trend * Whole colony size + Whole colony trend* | *7* | *1251.36* | *11.49* |
| *Whole colony size* | *4* | *1252.97* | *13.10* |
| *Sub-colony trend + Whole colony size* | *5* | *1254.40* | *14.53* |
| *Null* | *3* | *1259.83* | *19.96* |
| *Whole colony trend* | *4* | *1260.82* | *20.95* |
| *Sub-colony trend* | *4* | *1261.09* | *21.22* |
| *Sub-colony trend + Whole colony trend* | *5* | *1261.93* | *22.05* |

**S.6 AIC table for the proportion of new sites model:**

**Table S6.1 AIC table of generalised linear mixed-effects models with different fixed effect term structures to investigate the relationship between the proportion of new breeding sites established in a given year and sub-colony size and trend phase. Where two models were within 2 AIC units of one another we selected the model with the simplest model structure. The most supported model with the simplest model structure is shown in bold.**

| *Fixed effects structure* | Number of parameters | AIC | ∆AIC |
| --- | --- | --- | --- |
| *Sub-colony size + Sub-colony trend phase* | 6 | 712.07 | 0 |
| ***Sub-colony trend phase*** | **5** | **712.20** | **0.12** |
| *Sub-colony size* *+ Sub-colony trend phase + Whole colony trend phase* | 8 | 713.75 | 1.68 |
| *Sub-colony trend phase + Whole colony trend phase* | 7 | 714.26 | 2.19 |
| *Sub-colony size * Sub-colony trend phase* | 8 | 714.48 | 2.41 |
| *Whole colony trend phase* | 5 | 714.73 | 2.66 |
| *Sub-colony size + Whole colony trend phase* | 6 | 715.22 | 3.15 |
| *Sub-colony size* ** Sub-colony trend phase + Whole colony trend phase* | 10 | 717.06 | 4.98 |
| *Null* | 3 | 720.60 | 8.52 |
| *Sub-colony size* | 4 | 722.12 | 10.05 |

**S.7 AIC table for the proportion of new and reoccupied sites model:**

**Table S7.1 AIC table of generalised linear mixed-effects models with different fixed effect term structures to investigate the effect of trend phrase on the proportion of new breeding sites established and historical sites reoccupied in a given year. Where two models were within 2 AIC units of one another we selected the model with the simplest model structure. The most supported model with the simplest model structure is shown in bold.**

| *Fixed effects structure* | Number of parameters | AIC | ∆AIC |
| --- | --- | --- | --- |
| ***Occupation type * Sub-colony trend phase*** | **8** | **1387.97** | **0** |
| *Occupation type * Sub-colony trend phase + Whole colony trend phase* | 10 | 1390.82 | 2.84 |
| *Occupation type + Sub-colony trend phase* | 6 | 1425.95 | 37.98 |
| *Occupation type + Sub-colony trend phase + Whole colony trend phase* | 8 | 1428.29 | 40.32 |
| *Occupation type* | 4 | 1446.14 | 58.17 |
| *Occupation type + Whole colony trend phase* | 6 | 1446.90 | 58.93 |
| *Sub-colony trend phase* | 5 | 1461.79 | 73.82 |
| *Sub-colony trend phase + Whole colony trend phase* | 7 | 1464.33 | 76.36 |
| *Null* | 3 | 1480.81 | 92.84 |
| *Whole colony trend phase* | 3 | 1482.30 | 94.33 |

**S.8 AIC table for the proportion new sites model:**

**Table S8.1 AIC table of generalised linear mixed-effects models with different fixed effect term structures to investigate the effect of trend phrase on the quality of new breeding sites established and historical sites reoccupied in a given year. Where two models were within 2 AIC units of one another we selected the model with the simplest model structure. The most supported model with the simplest model structure is shown in bold.**

| *Fixed effects structure* | Number of parameters | AIC | ∆AIC |
| --- | --- | --- | --- |
| ***Occupation type * Sub-colony trend phase + Whole colony trend phase*** | **10** | **387.44** | **0** |
| *Occupation type * Sub-colony trend phase* | 8 | 394.29 | 6.85 |
| *Occupation type + Sub-colony trend phase + Whole colony trend phase* | 8 | 416.58 | 29.15 |
| *Occupation type + Sub-colony trend phase* | 6 | 422.52 | 35.08 |
| *Sub-colony trend phase + Whole colony trend phase* | 7 | 426.05 | 38.61 |
| *Sub-colony trend phase* | 5 | 436.94 | 49.51 |
| *Whole colony trend phase* | 6 | 439.79 | 52.36 |
| *Occupation type* | 4 | 445.66 | 58.22 |
| *Whole colony trend phase* | 5 | 449.58 | 62.14 |
| *Null model* | 3 | 453.25 | 65.81 |

**References:**

Birkhead, T. R., & Nettleship, D. N. (1987). Ecological relationships between Common Murres, Uria aalge, and Thick-billed Murres, Uria lomvia, at the Gannet Islands, Labrador. II. Breeding success and site characteristics. *Canadian Journal of Zoology*, *65*(7), 1630–1637. https://doi.org/10.1139/z87-252

Heubeck, M., Gear, S., & Harris, M. P. (2014). A photographic resurvey of seabird colonies on Foula, Shetland. *Scottish Birds*, *34*(3), 291–302.

Vincent, Q. V. (2011). ggbiplot: A ggplot2 based biplot. *R Package Version 0.55.* http://github.com/vqv/ggbiplot
